# Lead Phytochemicals for Anticancer Drug Development

**DOI:** 10.3389/fpls.2016.01667

**Published:** 2016-11-08

**Authors:** Sukhdev Singh, Bhupender Sharma, Shamsher S. Kanwar, Ashok Kumar

**Affiliations:** Department of Biotechnology, Himachal Pradesh UniversityShimla, India

**Keywords:** cancer, limitations of anticancer drugs, anticancer phytochemicals, druggability evaluation

## Abstract

Cancer is a serious concern at present. A large number of patients die each year due to cancer illnesses in spite of several interventions available. Development of an effective and side effects lacking anticancer therapy is the trending research direction in healthcare pharmacy. Chemical entities present in plants proved to be very potential in this regard. Bioactive phytochemicals are preferential as they pretend differentially on cancer cells only, without altering normal cells. Carcinogenesis is a complex process and includes multiple signaling events. Phytochemicals are pleiotropic in their function and target these events in multiple manners; hence they are most suitable candidate for anticancer drug development. Efforts are in progress to develop lead candidates from phytochemicals those can block or retard the growth of cancer without any side effect. Several phytochemicals manifest anticancer function *in vitro* and *in vivo*. This article deals with these lead phytomolecules with their action mechanisms on nuclear and cellular factors involved in carcinogenesis. Additionally, druggability parameters and clinical development of anticancer phytomolecules have also been discussed.

## Introduction

Cancer, a severe metabolic syndrome, is the leading cause of mortality and morbidity worldwide and the number of cases are continuously rising (Sharma et al., 2014; American Cancer Society, 2016). This disease ranks second in death cases after cardiovascular disorders in the developed nations (Mbaveng et al., 2011; Siegel et al., 2016). The cancer phenomenon is described by uncontrolled proliferation and dedifferentiation of a normal cell. Cancer cells have some marked features i.e., they tune out the signals of proliferation and differentiation, they are capable to sustain proliferation, they overcome the apoptosis, and they have power of invasion and angiogenesis. Sequential genetic alterations which produce genetic instabilities accumulate in the cell and a normal cell transforms into a malignant cell. These alterations include mutations in DNA repair genes, tumor suppressor genes, oncogenes and genes involve in cell growth & differentiation. These modifications are not just abrupt transitions but may take several years. Both external (e.g., radiations, smoking, pollution and infectious organisms) and internal factors (e.g., genetic mutations, immune conditions, and hormones) can cause cancer. Various types of cancer forms exist in human (Table 1); and the lung, breast and colorectal cancer being the most common forms (Ferlay et al., 2010). Among these the lung cancer is reported the most in men and the breast cancer in women (Horn et al., 2012). Several genes coordinate together for the growth & differentiation of a normal cell. In the cancer, one or group of these genes get altered and express aberrantly (Biswas et al., 2015). These genes can be targeted for the development of anticancer therapeutics. Modifications of epigenetic processes involved in cell growth and differentiation also lead to the development of a cancer. Therapeutic interventions which can reverse these epigenetic alterations may also be a promising option in anticancer drug discovery (Schnekenburger et al., 2014). Azacitidine, decitbine, vorinostat, and romidespin are exemplary epigenetic anticancer drugs in this regard.

**Table 1:** **Some epidemiological forms of cancer**.

Lung cancer	Breast cancer	Colorectal cancer
Lymphoma	Melanoma	Leukemia
Stomach cancer	Brain tumor	Prostate cancer
Cervical cancer	Ovary cancer	Kidney cancer
Liver cancer	Oral cavity cancer	Larynx cancer
Malignant ascites	Skin cancer	Uterus cancer
Pancreas cancer	Urinary bladder cancer	Thyroid cancer
Fibrosarcoma	Lymphosarcoma	

## Drugs for cancer treatment

Cancer treatment involves surgery of tumor, radiotherapy, and chemotherapy. Treatment method depends upon the stage and location of tumor. Chemotherapy involves cytotoxic and cytostatic drugs and proved to be very efficient when used in combination with other therapies. Authoritative chemotherapeutics include alkylating agents, topoisomerase inhibitors, tubulin acting agents and antimetabolites. Alkylating agents bind covalently with DNA, crosslink them and generate strand breaks. Carboplatin, cisplatin, oxaliplatin, cyclophosphamide, and melphalan are exemplary alkylating agents which work by causing such DNA damage. Doxorubicin and irinotecan are topoisomerase inhibitors and hinder DNA replication. Tubulin acting agents interrupt mitotic spindles and arrest mitosis. Paclitaxel, docetaxel, vinblastine and vincristine act on tubulins. Paclitaxel (C_47_H_51_NO_14_) has been proved an effective anticancer drug against most of the cancer types. It was isolated in 1967 from the endophytic fungi found in *Taxus brevifolia* bark (Carsuso et al., 2000). Paclitaxel targets tubulin and suppress microtubules detachment from the centrosomes. It induces multipolar divisions by forming abnormal spindles which bear additional spindle poles. This results in unnatural chromosome segregation and abnormal aneuploid daughter cells which go in apoptosis direction (Weaver, 2014). Antimetabolites stop nucleic acid synthesis and examples are methotrexate and 5-fluorouracil. Some other drugs with specific targets are also approved for the treatment of cancer. Bevacizumab inhibits vascular endothelial growth factor receptor and has been used to treat metastatic cancers (Van Meter et al., 2010). Rituximab targets CD20 in lymphoma, imatinib targets Bcr/Abl, gefitinib acts on epithelial growth factor receptor and bortezomib is approved as proteosome inhibitor (Murawski and Pfreundschuh, 2010).

## Limitations of available anticancer drugs

Existing anticancer drugs target rapidly dividing cells. Several cells in our body proliferate quickly under normal circumstances, viz. bone marrow cells, digestive tract cells and hair follicle cells. So these normal cells are also affected by the above mentioned drugs and serious side effects emerge. These side effects include myelosuppression (decreased production of blood cells), mucositis (inflammation of digestive tract lining), hair loss, cardiotoxicity, neurotoxicity and immunosuppression. Additionally, rapid elimination and widespread distribution of the introduced drug to the non-targeted organs by body system requires large dose of the drug which increases above mentioned side effects and also not economic. Another limitation is the resistance of cancer cells to the available drugs. Cancer cells undergo mutations and become resistant to the introduced drugs. Therefore, given the reality of unsatisfactory chemotherapy, innovations in treating cancer with fewer side effects are the trending research direction. An ideal anticancer drug will specifically be cytotoxic toward the cancer cells only and based on the research findings, phytochemicals & derivatives present in plants are promising option for the improved and less toxic cancer therapy. Bioactive phytochemicals possess diverse therapeutic functions (e.g., analgesic, anti-inflammatory, antitumor and many more). These phytomedicines cover an important portion of our current pharmaceutics (Table 2).

**Table 2:** **Medicinal products derived from plants**.

**Drug**	**Pharmacological function**
Aspirin	Analgesic, anti-inflammatory
Atropine	Pupil dilation
Bromelain	Anti-inflammatory
Colchicine	Anticancer
Digitoxin	Cardiotonic
Ginkgolides	Brain disorders
Harpogoside	Rheumatism
Hyoscyamine	Anti-cholinergic
Morphine	Analgesic
Podophyllotoxin	Anticancer
Quinine	Antimalarial
Reserpine	Anti-hypertensive
Salicin	Analgesic
Silymarin	Antihepatotoxic
Sitosterol	Prostate hyperplasia
Taxol	Anticancer
Vincristine and vinblastine	Anticancer
Tubocurarine	Mascular relaxation

## Plants as indispensable sources for anticancer phytochemicals

**Figure 1:**
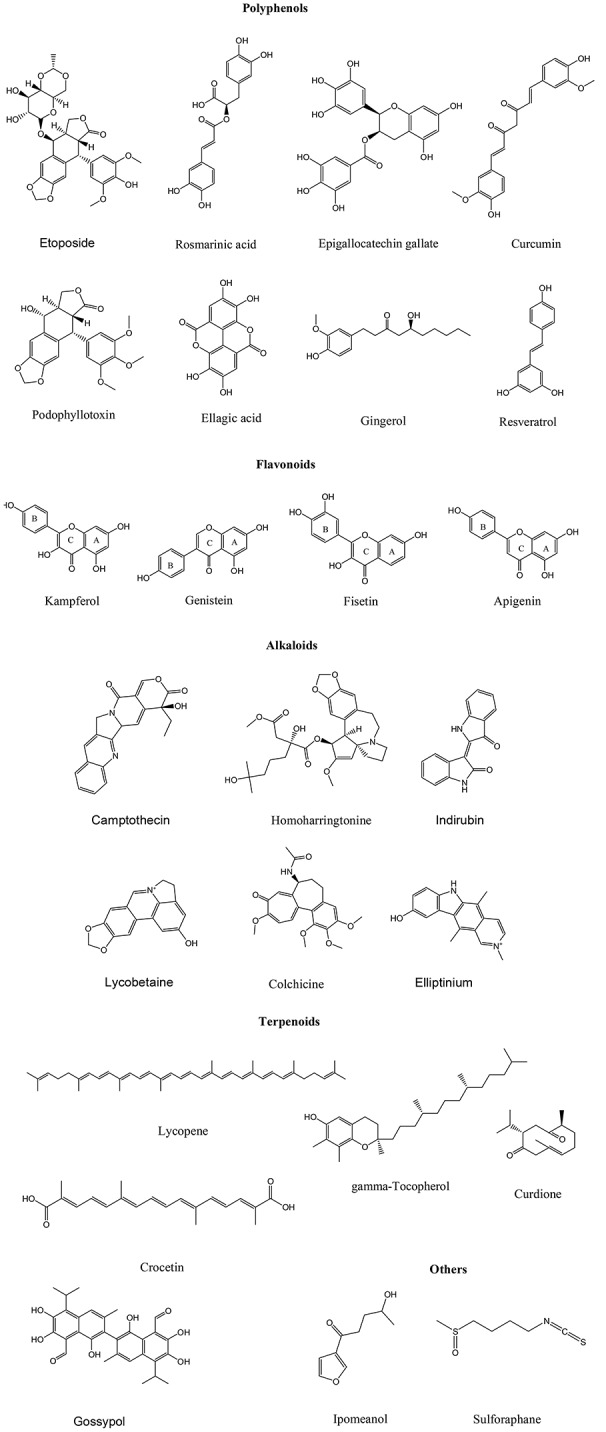
**Chemical structures of some anticancer phytochemicals**.

Plants and their formulations are in medicinal uses since ancient times. Various herbal preparations with different philosophies and cultural origins are used by folk medicine practitioners to heal kinds of diseases. Ayurveda, the ancient Vedic literature of India, is the science of good health and well-being (Behere et al., 2013). It is the collection of traditional and cultural philosophies to cure the diseases. Modern drug development program based on ayurveda concepts has gained wide acceptance in present healthcare system. Plant derived natural products are nontoxic to normal cells and also better tolerated hence they gain attention of modern drug discovery. Estimated figures reveal that plant kingdom comprises at least 250,000 species and only 10 percent have been investigated for pharmacological applications. Phytochemicals and their derived metabolites present in root, leaf, flower, stem and bark perform several pharmacological functions in human systems. Alkaloids, flavonoids, phenolics, tannins, glycosides, gums, resins and oils are such responsible elements (Singh et al., 2013). These elements or their altered forms have shown significant antitumor potential. Vinblastine, vincristine, taxol, elliptinium, etoposide, colchicinamide, 10-hydroxycamptothecin, curcumol, gossypol, ipomeanol, lycobetaine, tetrandrine, homoharringtonine, monocrotaline, curdione, and indirubin are remarkable phytomolecules in this regard (Figure 1).

## Anticancer functions of phytochemicals on animal and cellular models

Several plant species have been discovered to suppress the progression and development of tumors in cancer patients (Umadevi et al., 2013) and many phytochemicals have been identified as active constituents in these plant species (Table 3). Phytochemicals exert antitumor effects via distinct mechanisms. They selectively kill rapidly dividing cells, target abnormally expressed molecular factors, remove oxidative stress, modulate cell growth factors, inhibit angiogenesis of cancerous tissue and induce apoptosis. For example some polyphenols (e.g., resveratrol, gallocatechins), flavonoids (e.g., methoxy licoflavanone, alpinumisoflavone), and brassinosteroids (e.g., homocatasterone and epibrassinolide) exert anticancer effects through apoptosis induction (Heo et al., 2014; Wen et al., 2014). Curcumin, thymol, rosmarinic acid, β-carotene, quercetin, rutin, allicin, gingerol, epigallocatechin gallate, and coumarin perform anticancer functions through antioxidant mechanisms.

**Table 3:** **Phytochemicals which demonstrated effective tumor killing in various cancer types**.

**Phytochemical(s)**	**Cancer models suppressed**	**References**
Alexin B, Emodin (*Aloe vera*)	Leukemia, stomach cancer, neuroectodermal tumors	Elshamy et al., 2010
Allylmercaptocysteine, allicin (*Allium sativum*)	Colon cancer, bladder carcinoma	Ranjani and Ayya, 2012
Amooranin (*Amoora rohituka*)	Breast, cervical, and pancreatic cancer	Chan et al., 2011
Andrographolide (*Andrographis paniculata*)	Cancers of breast, ovary, stomach, prostate, kidney, nasopharynx malignant melanoma, leukemia	Geethangili et al., 2008
Ashwagandhanolide (*Withania somnifera*)	Cancers of breast, stomach, colon, lung, and central nervous system	Yadav et al., 2010
Bavachanin, corylfolinin, psoralen (*Psoralea corylifolia*)	Cancers of lung, liver, osteosarcoma, malignant ascites, fibrosarcoma, and leukemia	Wang et al., 2011
Berberine, Cannabisin-G (*Berberis vulgaris*)	Cancers of breast, prostate, liver, and leukemia	Elisa et al., 2015
Betulinic acid (*Betula utilis*)	Melanomas	Król et al., 2015
Boswellic acid (*Boswellia serrata*)	Prostate cancer	Garg and Deep, 2015
Costunolide, Cynaropicrine, Mokkolactone, (*Saussurea lappa*)	Intestinal cancer, malignant lymphoma, and leukemia	Lin et al., 2015
Curcumin (*Curcuma longa*)	Cancers of breast, lung, esophagus, liver, colon, prostate, skin and stomach	Perrone et al., 2015
Daidzein and genistein (*Glycine max*)	Cancers of breast, uterus, cervix, lung, stomach, colon, pancreas, liver, kidney, urinary bladder, prostate, testis, oral cavity, larynx, and thyroid	Li et al., 2012
Damnacanthal (*Morinda citrifolia*)	Lung cancer, sarcomas	Aziz et al., 2014
β-Dimethyl acryl shikonin, arnebin (*Arnebia nobilis*)	Rat walker carcinosarcoma	Thangapazham et al., 2016
Emblicanin A & B (*Emblica officinalis*)	Cancers of breast, uterus, pancreas, stomach, liver, and malignant ascites	Dasaroju and Gottumukkala, 2014
Eugenol, orientin, vicenin (*Ocimum sanctum*)	Cancers of breast, liver, and fibrosarcoma	Preethi and Padma, 2016
Galangin, pinocembrine, acetoxy chavicol acetate (*Alpinia galangal*)	Cancers of lung, breast, digestive systems, prostate, and leukemia	Sulaiman, 2016
Gingerol (*Zingiber officinale*)	Cancers of ovary, cervix, colon, rectum, liver, urinary bladder, oral cavity, neuroblastoma, and leukemia	Rastogi et al., 2015
Ginkgetin, ginkgolide A & B (*Ginkgo biloba*)	Glioblastoma multiforme, hepatocarcinoma, ovary, prostate, colon, and liver	Xiong et al., 2016
Glycyrrhizin (*Glycyrrhiza glabra*)	Lung cancer, fibrosarcomas	Huang et al., 2014
Gossypol (*Gossypium hirsutum*)	Cancers of breast, esophagus, stomach, liver, colon, pancreas, adrenal gland, prostate, urinary bladder, malignant lymphoma & myeloma, brain tumor, and leukemia	Zhan et al., 2009
Kaempferol galactoside (*Bauhinia variegata*)	Cancers of breast, lung, liver, oral cavity, larynx, and malignant ascites	Tu et al., 2016
Licochalcone A, Licoagrochalcone (*Glycyrrhiza glabra*)	Cancers of prostate, breast, lung, stomach, colon, liver, kidney, and leukemia	Zhang et al., 2016
Lupeol (*Aegle marmelos*)	Breast cancer, lymphoma, melanoma, and leukemia	Wal et al., 2015
Nimbolide (*Azadirachta indica*)	Colon cancer, lymphoma, melanoma, leukemia, and prostate cancer	Wang et al., 2016
Panaxadiol, panaxatriol (*Panax ginseng*)	Cancers of breast, ovary, lung, prostate, colon, renal cell carcinoma, leukemia, malignant lymphoma, and melanoma	Du et al., 2013
Plumbagin (*Plumbago zeylanica*)	Cancers of breast, liver, fibrosarcoma, leukemia, and malignant ascites	Yan et al., 2015
Podophyllin & podophyllotoxin (*Podophyllum hexandrum*)	Cancers of breast, ovary, lung, liver, urinary bladder, testis, brain, neuroblastoma, and Hodgkin's disease	Liu et al., 2015
Psoralidin (*Psoralea corylifolia*)	Stomach and prostate cancer	Pahari et al., 2016
Sesquiterpenes and diterpenes (*Tinospora cordifolia*)	Lung, cervix, throat, and malignant ascites	Gach et al., 2015
6-Shogaol (*Zingiber officinale*)	Ovary cancer	Ghasemzadeh et al., 2015
Skimmianine (*Aegle marmelos*)	Liver tumors	Mukhija et al., 2015
Solamargine, solasonine (*Solanum nigrum*)	Cancers of breast, liver, lung, and skin	Al Sinani et al., 2016
Thymoquinone (*Nigella sativa*)	Colon, breast, prostate, pancreas, uterus, malignant lymphoma, ascites, melanoma, and leukemia	Fakhoury et al., 2016
Ursolic acid and oleanolic acid (*Prunella vulgaris*)	Cancers of breast, cervix, lung, oral cavity, esophagus, stomach, colon, thyroid, malignant lymphoma, intracranial tumors, and leukemia	Wozniak et al., 2015
Ursolic acid (*Oldenlandia diffusa*)	Cancers lung, ovary, uterus, stomach, liver, colon, rectum, brain, lymphosarcoma, and leukemia (Al Sinani et al., 2016)	Wozniak et al., 2015
Vinblastine, vincristine (*Catharantus roseus*)	Cancers of breast, ovary, cervix, lung, colon, rectum, testis, neuroblastoma, leukemia, rhabdomyosarcoma, malignant lymphoma, and hodgkin's disease	Keglevich et al., 2012
Viscumin, digallic acid (*Viscum album*)	Cancers of breast, cervix, ovary lung, stomach, colon, rectum, kidney, urinary bladder, testis, fibrosarcoma, melanoma	Bhouri et al., 2012
Withaferin A, D (*Withania somnifera*)	Cancers of breast, cervix, prostate, colon, nasopharynx, larynx, and malignant melanoma	Lee and Choi, 2016

Anticancer drug development involves *in vitro* cytotoxicity on cancer cells, *in vivo* confirmation, and clinical trial evaluation. Assessment of cytotoxicity toward cancer cell lines is a trending strategy for the discovery of anticancer agents. Cell viability assessment of cancer cells is a high throughput screening method through which numerous compounds can be screened in a short period of time. Several such phytochemicals have been discovered from plants and dietary supplements (Table 4). Crude phytochemical extracts also suppress the viability of cancer cells (Table 5).

**Table 4:** **Phytochemicals which are cytotoxic against cancer cell lines**.

**Phytochemical(s)**	**Cell line**	**References**
Actein (*Actaea racemosa*)	HepG2 (liver cancer), MCF7 (breast cancer)	Einbonda et al., 2009
β-Aescin (*Aesculus hippocastanum*)	HT29 (colorectal adenocarcinoma)	Zhang et al., 2010
Allylmercaptocysteine, allicin (*Allium sativum*)	HeLa (cervix cancer), L5178Y (lymphoma), MCF7	Karmakar et al., 2011
Amooranin (*Amoora rohituka*)	MCF7, CEM (lymphocytic leukemia)	Chan et al., 2011
Asiatic acid (*Centella asiatica*)	EAC (ehrlich ascites carcinoma), DLA (dalton's lymphoma ascites), SK-MEL-2 (melanoma), U-87-MG (glioblastoma), B16F1 (melanoma), MDA-MB-231 (breast adenocarcinoma)	Heidari et al., 2012
Baicalein (*Scutellaria baicalensis*)	LXFL-529L (lung cancer)	Zhou et al., 2008
Carnosic acid, rosmarinic acid (*Rosmarinus officinalis*)	A549 (lung cancer), SMMC7721 (liver cancer), A2780 (ovary cancer)	Tai et al., 2012
β-Caryophyllene, α-zingiberene (*Peristrophe bicalyculata*)	MCF7, MDA-MB-468	Ogunwande et al., 2010
Cinnamaldehyde (*Cinnamomum zeylanicum*)	HeLa, HCT116 (colorectal carcinoma), HT29	Koppikar et al., 2010
Clausenalansamide A and B (*Clausena lansium*)	SGC-7901 (gastric cancer), HepG2, A549	Maneerat et al., 2011
Corydine, salutaridine (*Croton macrobotrys*)	NCI-H460 (lung cancer), K5662 (leukemia)	Motta et al., 2011
Conyzapyranone A and B (*Conyza Canadensis*)	HeLa, A431 (epidermoid carcinoma), MCF7	Csupor-Loffler et al., 2011
Costunolide, tulipinolide, liriodenine, germacranolide (*Liriodendron tulipifera*)	KB (oral cancer), HT29	Wang et al., 2012
Crocin, picrocrocin, crocetin, and safranal (*Crocus sativus*)	S-180 (sarcoma), EAC, DLA, Tca8113 (oral cancer), and HCT116	Bakshi et al., 2009
Dioscin (*Dioscorea colletti*)	HepG2, SGC-7901	Hu et al., 2011
Epicatechin, procyanidin B2, B4 (*Litchi cinensis*)	MCF7	Bhoopat et al., 2011
Gallic acid (*Leea indica*)	EAC	Raihan et al., 2012
Harringtonines (*Cephalotaxus harringtonia*)	P388 (blood cancer), L-1210 (blood cancer)	Jin et al., 2010
Hydroxycinnamoyl ursolic acid (*Vaccinium macrocarpon*)	MCF7, ME180 (cervical cancer), and PC3 (prostate cancer)	Kondo et al., 2011
Leachianone A (*Radix sophorae*)	HepG2	Long et al., 2009
Lectin (*Hibiscus mutabilis*)	HepG2, MCF7	Lam and Ng, 2009
Naphthoquinones (*Tabebuia avellanedae*)	EAC, DU145 (prostate cancer)	Yamashita et al., 2009
Niazinine A (*Moringa oliefera*)	AML (blood cancer), HepG2	Khalafalla et al., 2011
Pectolinarin (*Cirsium japonicum*)	S180, H22 (liver cancer)	Yin et al., 2008
Piperine, piperidine (*Piper nigrum*)	B16F10 (melanoma)	Liu et al., 2010
Platycodin D (*Platycodon grandiflorum*)	A549, HT29	Shin et al., 2009
Plumbagin (*Plumbago zeylanica*)	PC3, A549, NB4 (blood cancer), A375 (skin cancer), SGC7901	Checker et al., 2010
Pyrogallol (*Emblica officinalis*)	L929	Baliga and Dsouza, 2011
Tylophorine (*Tylophora indica*)	A549, DU145, ZR751 (breast cancer)	Kaur et al., 2011
Withaferin A (*Withania somnifera*)	MCF7, MDA-MB-231	Hahm et al., 2011

**Table 5:** **Plants whose phytochemical preparation is cytotoxic toward cancer cell lines**.

**Plant**	**Cancer cell line(s)**	**GI_50_ (μg/ml)^#^**	**References**
*Abelia triflora* (leaf)	A549	1000	Al-Taweel et al., 2015; Rafshanjani et al., 2015
*Abutilon indicum* (leaf)	A549	85.2	Kaladhar et al., 2014
*Amphipterygium adstringens* (bark)	OVCAR3 (ovary adenocarcinoma)		Garcia et al., 2015
*Annona crassiflora* and *A. coriacea* (leaf and seed)	UA251 (glioma), UACC62 (lymphoid melanoma), MCF7, NCIH460 (lung cancer), 786-0 (renal carcinoma), HT29, NCIADR-RES (ovary cancer), OVCAR, K562 (myeloid leukemia)	≈8.9	Formagio et al., 2015
*Argemone gracilenta*	M12.C3F6 (B-cell lymphoma) RAW264.7 (leukemia) HeLa	46.2 64.45 78.87	Leyva-Peralta et al., 2015
*Camellia sinensis* (black tea)	MCF-7	0.0125	Konariková et al., 2015
*Catharanthus roseus Emblica officinalis* (fruit)	HCT116	46.21 35.21	Bandopadhyaya et al., 2015
*Centaurea kilaea*	MCF7 HeLa PC3	60.7 53.07 73.92	Selvarani et al., 2015; Sen et al., 2015
*Chamaecyparis obtuse* (leaf)	HCT116	1.25	Kim et al., 2015
*Chresta sphaerocephala* (leaf and stem)	OVCAR3 786-0 U251	50.4 72.3 77.6	Da Costa et al., 2015
*Cichorium intybus*	MCF7	429	Gospodinova and Krasteva, 2015
*Croton sphaerogynus* (leaf)	U251, MCF7, NCIH460	–	dos Santos et al., 2015
*Curcuma longa* (rhizome)	A549	207.3	Shah et al., 2015
*Elaeocarpus serratus* (leaf)	MCF7	227	Sneha et al., 2015
*Fraxinus micrantha*	MCF7	18.95	Kumar and Kashyap, 2015
*Harpophyllum caffrum* (leaf)	HCT116 A549 MCF7 HepG2	29 28.8 21 29	El-Hallouty et al., 2015
*Holarrhena floribunda* (leaf)	MCF7 HT29 HeLa	126.7 159.4 106.7	Badmus et al., 2015
*Ipomoea quamoclit* (leaf)	CNE1 (nasopharynx carcinoma) HT29 MCF7 HeLa	18 18 31 33	Ho et al., 2015
*Ipomoea pestigridis* (leaf)	HepG2	155.2	Begum et al., 2015; Kabbashi et al., 2015
*Justicia tranquibariensis* (roots)	HeLa	>200	Jiju, 2015; Nair et al., 2015
*Nardostachys jatamansi* (roots and rhizome)	MCF7 MDA-MB-231	58.01 23.83	Chaudhary et al., 2015
*Piper cubeba* (seed)	MCF7 MDA-MB-468	22.31 21.84	Graidist et al., 2015
*Piper pellucidum* (leaf)	HeLa	2.85	Wahyu et al., 2013
*Premna odorata* (bark)	HCT116 MCF7 A549 AA8	8.58 8.42 11.42 18.09	Tantengco and Jacinto, 2015
*Psidium guajava* (leaf)	EAC HeLa MDA-MB-231 MG-263	25 49.5 6.19 6.19	Shakeera and Sujatha, 2015
*Robinia pseudoacacia*	C6 (glioma), MCF, T47D (breast cancer), A549	-	Bhat et al., 2015
*Rubus fairholmianus* (root)	Caco2	20	George et al., 2015
*Sansevieria liberica* (root)	HeLa HCT116 THP1 (leukemia)	23 22 18	Akindele et al., 2015
*Sideritis syriaca* (leaf)	MCF7	100	Yumrutas et al., 2015
*Solanum nigrum*	HeLa SiHa (cervix carcinoma) C33A (cervix carcinoma)	250 87.5 100	Paul et al., 2015
*Theobroma cacao* (leaf)	MCF7	41.4	Baharum et al., 2014
*Trapa acornis* (shell)	SKBR3 (breast cancer) MDA-MB-435	56.07 31.60	Pradhan and Tripathy, 2014
*Tridax procumbens*	A549, HepG2	-	Priya and Srinivasa, 2015
*Triumfetta welwitschii*	Jurkat T cells (T-cell leukemia)	–	Batanai and Stanley, 2015
*Vernonia amygdalina* (leaf)	HL60 SMMC7721 A549 MCF7 SW480 (colorectal adenocarcinoma)	5.58 8.74 10.97 9.51 8.84	Iweala et al., 2015
*Vinca major* (aerial parts)	HEp-2 (HeLa derived)	228	Singh et al., 2014

# *Concentration of plant preparation at which 50% growth inhibition of cells found*.

## Molecular targets and mechanisms of action of anticancer phytochemicals

The exact mechanism by which phytochemicals perform anticancer functions is still a topic of research. They exert wide and complex range of actions on nuclear and cytosolic factors of a cancer cell. They can directly absorb the reactive oxygen species (ROS) or promote activities of antioxidant enzymes (e.g., superoxide dismutase, glutathione and catalase) in a transformed cell. A phytomolecule can suppress malignant transformation of an initiated pre-neoplastic cell or it can block the metabolic conversion of the pro-carcinogen. Also, they can modulate cellular and signaling events involved in growth, invasion and metastasis of cancer cell. Ellagic acid of pomegranate induces apoptosis in prostate and breast cancer cells, and inhibits metastasis processes of various cancer types. Epigallocatechin gallate (EGCG) suppresses the activity of ornithine decarboxylase, the enzyme which signals the cell to proliferate faster and bypass apoptosis. Luteolin obstructs epithelial mesenchymal transition. Flavanones, isoflavones and lignans prevent binding of the estrogen to the cancer cells and reduce their proliferation. Reduction of inflammation processes through suppression of nuclear factor- kappa B (NF-κB) family transcription factors is an additional mechanism. Curcumin, bilberries anthocyanins, EGCG, caffeic acid and derivatives and quercetin act via NF-κB signaling. Besides these mechanisms, anticancer phytomolecules targets several other signaling molecules/ pathways also to reduce the growth and metastasis of cancer cells.

Apigenin, the flavone found in parsley, celery, and chamomile targets leptin/leptin receptor pathway and induces apoptosis in lung adenocarcinoma cells. It induces caspase dependent extrinsic apoptosis in human epidermal growth factor receptor 2 (HER-2) over expressing BT-474 breast cancer cells through inhibition of signal transducer and activator of transcription 3 (STAT3) signaling (Seo et al., 2015). Curcumin, the polyphenol of *Curcuma longa* slows down the growth of human glioblastoma cells by modulating several molecular components. It upregulates the expressions of p21, p16, p53, early growth response protein 1 (Egr-1), extracellular signal regulated kinase (Erk), c-Jun-N-terminal kinase (JNK), ElK-1 (member of ETS oncogenic family), Bcl-2 associated X protein (Bax) and Caspase- 3, 8, 9 proteins; and downregulates the levels of B cell lymphoma 2 (Bcl-2), mechanistic target of rapamycin (mTOR), p65, B cell lymphoma extra-large protein (Bcl-xL), protein kinase B (Akt), epidermal growth factor receptor (EGFR), NF-κB, cell division cycle protein 2 (cdc-2), retinoblastoma protein (pRB), cellular myelocytomatosis oncogenes (c-myc) and cyclin D1 proteins (Vallianou et al., 2015).

Crocetin, the carotenoid present in *Crocus sativus* and *Gardenia jasminoides* act on GATA binding protein 4 and MEK-ERK1/2 pathway and protect against cardiac hypertrophy (Cai et al., 2009). Cyanidin glycosides from red berries execute antioxidant and anticancer functions through various mechanisms. In colon cancer cells, they suppress the expressions of inducible nitric oxide synthase (iNOS) and cyclooxygenase-2 (COX-2) genes and inhibit mitogen induced metabolic pathways (Serra et al., 2013). In human vulva cells, they target EGFR and in breast cancer cells they block the ErbB2/cSrc/FAK pathway and prevent their metastasis (Xu et al., 2010). EGCG, the catechin polyphenol of tea exert anticancer effects through multiple mechanisms. It blocks NF-κB activation, Bcl-2 and COX-2 expression in prostate carcinoma cells and induces apoptosis. In bladder and lung carcinoma cells, it inhibits matrix metallopeptidase-9 (MMP-9) expression (Singh et al., 2011). In head & neck carcinoma cells it suppresses the production of vascular endothelial growth factor (VEGF). In fibrosarcoma cells it prevents the Erk phosphorylation and activity of MMP-2 & 9. And, in gastric carcinoma cells it suppresses the Erk, JNK, and MMP-9 expressions (Luthra and Lal, 2016).

Fisetin and hesperetin cause cell cycle arrest in human acute promyelocytic leukemia HL-60 cells by altering several signaling networks, viz. mitogen activated protein kinases (MAPK), NF-κB, JAK/STAT, PI3K/Akt, Wnt, and mTOR pathways (Adan and Baran, 2015). Genistein, the isoflavone of soybean exerts its anticancer effects via inhibition of NF-κB and Akt signaling pathways (Li et al., 2012). Gingerol targets the Erk1/2/JNK/AP-1 signaling and induces caspase-dependent apoptosis in colon cancer cells (Radhakrishnan et al., 2014). Kaempferol acts on proto-oncogene tyrosine protein kinase (Src), Erk1/2 and Akt pathways in pancreatic cancer cells and retard their growth & migration (Lee and Kim, 2016). Similarly, lycopene targets PI3K/Akt pathway in pancreatic cancer cells. It prevents gastric carcinogenesis by inhibiting Erk and Bcl-2 signaling (Kim and Kim, 2015). It activates antioxidant enzymes (e.g., GST, GSH, and GPx) in the cancer cells and removes oxidative damage produced by the carcinogen. Rosmarinic acid reduces COX-2 activity and Erk phosphorylation in colon cancer cells (Hossan et al., 2014). In breast cancer cells, rosmarinic acid reduces the activity of DNA methyl transferase and interfere RANKL/RANK/OPG networks. Also, it targets PKA/CREB/MITF pathway and NF-κB activation in melanoma and leukemia U938 cells respectively (Hossan et al., 2014).

Calcitriol inhibits prostaglandins, COX-2, NF-κB, and VEGF signaling and prevents angiogenesis of cancer cells (Diaz et al., 2015). Tocotrienols and γ-tocopherol hinder PI3K/Akt and Erk/MAPK pathways (Sylvester and Ayoub, 2013). Colchicine upregulates dual specificity phosphatase 1 (DUSP1) gene in gastric carcinogenesis (Lin et al., 2016). It also prevents the growth of hepatocellular carcinoma cells through upregulation of A-kinase anchoring protein 12 (AKAP12) and transforming growth factor beta-2 (TGF-β2) proteins (Kuo et al., 2015). Podophyllotoxin blocks the growth of MCF-7 breast cancer cells by altering checkpoint kinase 2 (Chk-2) signaling pathway (Zilla et al., 2014). Podophyllotoxin also promotes apoptosis in non-small cell lung carcinoma cells through ER stress, autophagy and cell cycle arrest (Choi et al., 2015). Vinblastine and taxol target activator protein 1 (AP-1) signaling pathways (Flamant et al., 2010). Resveratrol arrest carcinogenesis by multiple mechanisms, viz. upregulation of p53 and BAX proteins and downregulation of NF-κB, AP-1, hypoxia induced factor 1α (HIF-1α), MMPs, Bcl-2, cytokines, cyclins, cyclin dependent kinases (CDKs) and COX-2 proteins (Varoni et al., 2016).

These phytomolecules act on epigenetic elements also. DNA methylation, histone modifications and miRNAs expression are important epigenetic processes which involve in cancer initiation and progression. Phytomolecules reduce the activities and expression of DNA methyl transferases (DNMTs), histone deacetylases (HDACs) and histone methyl transferases (HMTs) and increase promoter demethylation in various cancer models (Thakur et al., 2014).

## Process development for purification of anticancer phytochemicals

**Figure 2:**
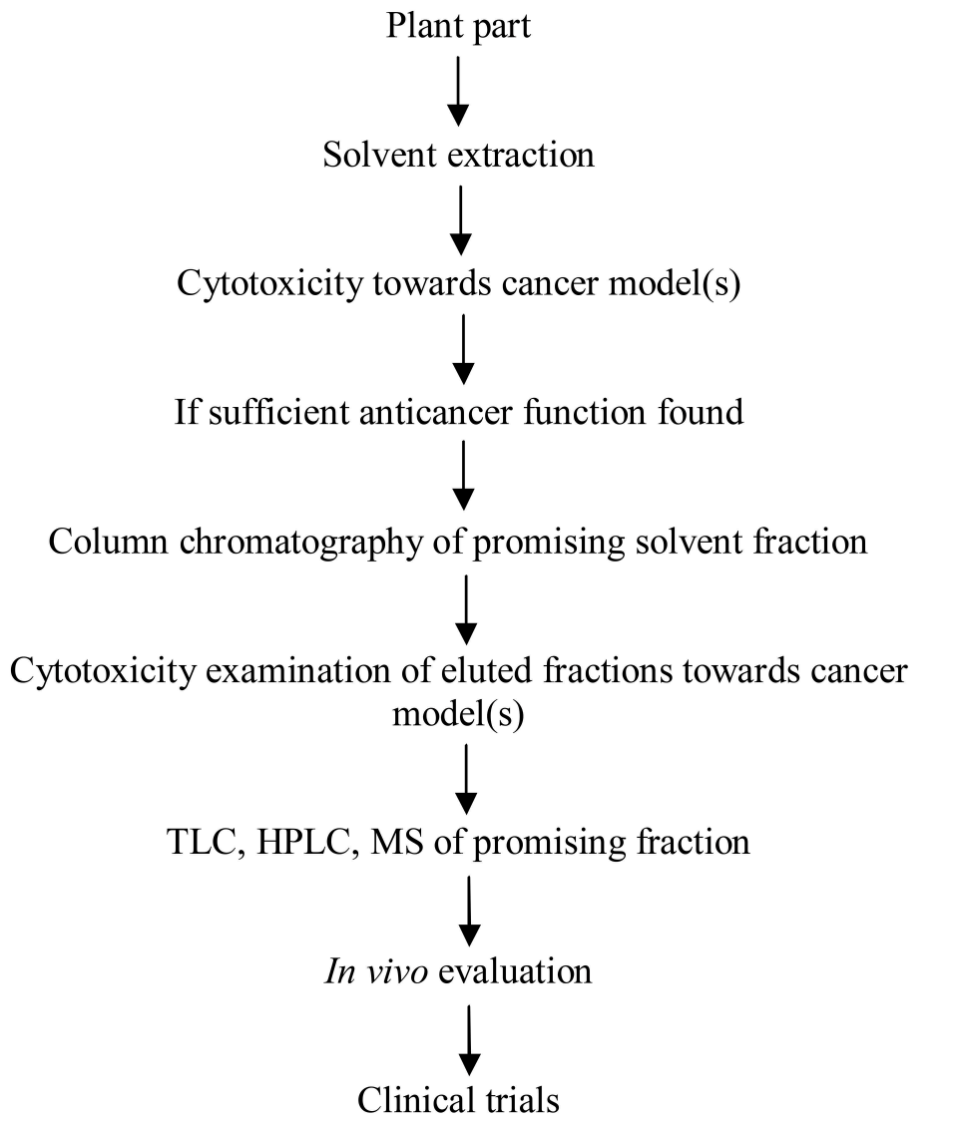
**Development of the bioactive phytomolecule(s) into an anticancer product**.

Therapeutic efficacy of any medicinal plant depends upon the quality and quantity of the active phyto-constituent(s), which vary with latitude, altitude, climate and season. Different parts of a plant may possess different level of pharmacological activity. Additive or synergistic effects of bioactive phyto-constituents might be responsible for the concerned pharmacological function rather than the purified one. These bioactive phyto-constituents can be developed in antitumor therapeutic entities but they demand intense effort. Several approaches can be used to purify these pharmacologically active phytochemicals. These include isolation and assay, combinatorial chemistry and bioassay-guided fractionation. Bioassay guided fractionation involves the separation of bioactive phytochemicals from a mixture of compounds using various analytic techniques based on biological activity testing. The process begins with the testing of natural extract with the confirmed bioactivity. The active extract is fractionated on suitable matrices, eluted fractions are tested for bioactivity and active fractions are examined by various analytic techniques, viz. thin layer chromatography (TLC), HPLC, FTIR, and Mass spectroscopy (MS). This approach can also be used to purify antimicrobial, antilipolytic and antioxidant compounds (Figure 2). Solvents should be used in an increasing polarity order. Silica, Sephadex, Superdex, or any other suitable matrix can be used for fractionation. Vanilline-sulfuric acid can be used as dyeing reagent for the detection natural compounds. The procedures may be modified but purity and quantity of the bioactive molecule must be high as much as possible and this can be achieved by using good quality of solvents, experimental careful handling and efficiency of the procedure. After purification, molecule(s) must be examined *in vivo* for the anticancer effects. If a promising tumor killing is achieved by the molecule then other parameters i.e., safety and adverse effects, dose concentration, pharmacokinetics, drug interactions etc. must be explored for the drug molecule.

## Druggability evaluation of natural products

Bioactive functional leads of natural origins can be used as original or must be converted into the druggable forms. Outrageous cost and reduced number of new drug approvals are serious challenges to the new drug discovery. The major task in plant-based drug discovery is the selection of right candidate species of pharmacological activity. This identification can be achieved using several approaches, viz. random selection, ethnopharmacology, codified systems of medicine, ayurvedic classical texts or zoopharmacognosy. Analysis of ayurvedic attributes of ancient healing formulations for the initial selection and bioactivity guided fractionation of the identified plant is a strategy of worth with greater success rate and reduced cost, time, and toxicity parameters. The *Bhavprakash* is an important ayurvedic text for the cancer prevention. The botanic candidate can be selected on the basis of ethnomedicine uses also. These plant species can be safer than the species with no history of human use. Another challenge with plant based medicinal products is their poor bioavailability due to excessive degradation by drug metabolic enzymes. The chemical entities of plants have greater steric complexity, greater number of chiral centers, greater number of hydrogen bond donors and acceptors, diverse in aromaticity and higher molecular mass and rigidity which pose strings of challenges in their development into a clinical product.

Therapeutic phytomolecules and dietary supplements of natural origins are governed by Food and Drug Administration (FDA, USA) and Dietary Supplement Health and Education Act 1994 respectively for their pharmacological effects and safety issues. Despite having desired bioactivity and ADMET profile (absorption, distribution, metabolism, excretion, and toxicity), most of the chemical entities of natural origin do not fulfill Lipinski's “rule of five” the drug likeness criteria according to which, a candidate should have less than 500 Da molecular weight, less than 5 hydrogen bond donors, less than 10 hydrogen bond acceptors and less than 5 partition coefficient (log*P*) for being work as a drug molecule. These rules are aimed to achieve highest bioavailability. Nonetheless some breakthrough natural products for e.g., paclitaxel would have never become a drug based on these Lipinski's conventions. So there is an essential need to develop suitable druggability standards for the compounds/formulations of natural origins. Various coating materials, micelles, liposomes, phospholipid complexes and nano-materials may be used to enhance the bioavailability of phytomedicines.

## Conclusion

The world is moving toward natural products due to their low cost and reliability over side effects resulted from existing drugs. Researchers are intensifying their efforts for the development of phytopharmaceuticals against severe metabolic syndromes including cancer. Bioactive phytochemicals/formulations are potential leads for the development of safer anticancer drugs. Several plants and their constitutive phytochemicals have been screened for this purpose but only a very few have reached up to the clinical level. Anticancer phytochemicals described in this article must be further researched in clinical trials for their effectiveness and toxicological documentation. They must be developed as druggable forms with sufficient bioavailability. Moreover, we know that a traditional herbal preparation has greater medicinal effect than the same phytochemical/molecule taken in a pure form. So therapeutic intervention based upon the combination of anticancer molecules may give potent and effective therapeutic results.

India is the largest producer of medicinal plants and many of the current health care products. Substantial scientific work has been done on Indian plants and their products for the treatment of frightful diseases. They must be explored for anticancer potential. Furthermore, medicinal attributes put the medicinal plants in high demand and draw the attention of world in risking their biodiversity. Due to increasing demand and deforestation, over exploitation of the medicinal plants continues worldwide with time. Thus, some of the medicinal plants may become extinct soon. Therefore, medicinal plants need their conservation also. Germplasm preservation, viable seed preservation and cryopreservation are promising strategies for the same. In final words, this review can help healthcare pharmacy to explore values of medicinal plants to treat malignancies.

## Author contributions

All authors listed, have made substantial, direct and intellectual contribution to the work, and approved it for publication.

### Conflict of interest statement

The authors declare that the research was conducted in the absence of any commercial or financial relationships that could be construed as a potential conflict of interest.
